# Association between tertiary lymphoid structures and clinical outcomes in cancer patients treated with immune checkpoint inhibitors: an updated meta-analysis

**DOI:** 10.3389/fimmu.2024.1385802

**Published:** 2024-06-27

**Authors:** Lingli Li, Yusheng Guo, Bingxin Gong, Sichen Wang, Maggie Meijia Wang, Peng Sun, Shanshan Jiang, Lian Yang

**Affiliations:** ^1^ Department of Radiology, Union Hospital, Tongji Medical College, Huazhong University of Science and Technology, Wuhan, China; ^2^ Hubei Key Laboratory of Molecular Imaging, Wuhan, China; ^3^ School of Life Science and Technology, Harbin Institute of Technology, Harbin, China; ^4^ Wuhan Britain-China School, Wuhan, China; ^5^ Clinical & Technical Support, Philips Healthcare, Beijing, China

**Keywords:** cancer, immunotherapy, meta-analysis, prognosis, tertiary lymphoid structures

## Abstract

**Background:**

Although numerous studies have reported the association between tertiary lymphoid structures (TLSs) and clinical outcomes in cancer patients treated with immune checkpoint inhibitors (ICIs), there remains a lack of a newer and more comprehensive meta-analysis. The main objective of this study is to explore prognostic biomarkers in immunotherapy-related patients, through analyzing the associations between tertiary lymphoid structures (TLSs) and clinical outcomes in cancer patients treated with ICIs, so as to investigate their prognostic value in cancer patients treated with ICIs.

**Methods:**

A comprehensive search was conducted until February 2024 across PubMed, Embase, Web of Science, and the Cochrane Library databases to identify relevant studies evaluating the association between tertiary lymphoid structures and clinical outcomes in cancer patients treated with ICIs. The clinical outcomes were overall survival (OS), progression‐free survival (PFS), and objective response rate (ORR).

**Results:**

Thirteen studies were incorporated in this meta-analysis, among which nine evaluated the prognostic value of TLSs. The results showed the high levels of TLSs predicted a significantly prolonged OS (pooled HR = 0.35, 95% CI: 0.24–0.53, p < 0.001) and PFS (pooled HR = 0.47, 95% CI: 0.31–0.72, p < 0.001), while lower ORR (pooled OR = 3.78, 95% CI: 2.26–6.33, p < 0.001) in cancer patients treated with ICIs.

**Conclusion:**

Our results indicated that high levels of TLSs could predict a favorable prognosis for cancer patients treated with ICIs and have the potential to become a prognostic biomarker of immunotherapy-related patients.

## 1 Introduction

Recently, exciting progress in cancer immunotherapy has ushered in a new era in cancer treatment, especially in the therapeutic domain of numerous solid tumors (1). The immune checkpoint inhibitors (ICIs) mainly included anti-cytotoxic T-lymphocyte-associated protein 4 (CTLA-4) and anti-programmed cell death-1/programmed death ligand-1 (PD-1/PD-L1) (2). ICIs have improved patient survival in solid tumors, such as melanoma, non-small cell lung cancer, metastatic urinary tract carcinoma, hepatocellular carcinoma, gastric cancer (3–6). However, not all cancer patients benefit from immunotherapy. For instance, only approximately 5% of patients with metastatic triple-negative breast cancer obtain a positive response to PD-1/PD-L1 blockade (7, 8). Therefore, investigation of the corresponding biomarkers predicting immunotherapy response is of great significance for cancer patients.

Tertiary lymphoid structures (TLSs) are ectopic lymphoid organs formed in nonlymphoid tissues during chronic inflammation and tumorigenesis, which include B cells and T cells (9). The immune cells present in TLSs enhance the presentation of tumor antigens, amplify signaling through cytokines, and activate CD8+ T cells to target and destroy tumor cells (10, 11). TLSs play a crucial role as the focal points for triggering and sustaining both local and systemic T and B cell responses to tumors. TLSs identified from several solid tumors have been demonstrated to be correlated with the outcomes in cancer patients treated with ICIs (11–19). In general, the presence or a higher density of TLSs is an indicator of a favorable prognosis in cancer patients treated with ICIs (11–16, 18). Notably, a few studies also reported TLSs presence was not significantly associated with either PFS or OS in cancer patients treated with ICIs, such as head and neck squamous cell carcinoma and colorectal cancer (20, 21).

However, there is a lack of uniform standards for TLSs evaluation. Various studies have adopted different criteria, with some categorizing TLSs as either high or low density (22–24), and others using the mere existence or non-existence of TLSs as a benchmark for evaluation (23, 25). Additionally, the degree of TLSs maturity is a factor considered in some studies (26). The diversity in these classification methods may influence the prognostic predictive power associated with TLSs. Therefore, it is necessary to conduct a newer and more comprehensive meta-analysis to explore the association between tertiary lymphoid structures (TLSs) and clinical outcomes in cancer patients treated with ICIs. The main objective of this study is to explore prognostic biomarkers in immunotherapy-related patients, through analyzing the associations between tertiary lymphoid structures (TLSs) and clinical outcomes in cancer patients treated with ICIs, so as to investigate their prognostic value in cancer patients treated with ICIs.

## 2 Methods

### 2.1 Search strategy

This meta-analysis and systematic review adhered to the Preferred Reporting Items for Systematic Reviews and Meta‐Analyses guidelines (27) and the protocol for the analysis was registered prospectively in PROSPERO (CRD42024504778). A comprehensive search was conducted on multiple databases including PubMed, Web of Science, the Cochrane Library, and Embase to identify relevant studies published until February 2024. The search terms included tertiary lymphoid structures (TLSs), tertiary lymphoid organ (TLO), tertiary lymphoid tissue (TLT), ectopic lymphoid-like structures (ELSs), cancer/tumor/solid tumor, immunotherapy, ICI, immune checkpoint inhibitor and prognosis, prognostic or survival outcome. The exact search query is provided as follows to allow reproducibility: (TLSs OR TLO OR TLT OR ELSs) AND (cancer OR tumor OR solid tumor) AND (prognosis OR prognostic OR survival outcome). Two researchers (LLL and YSG) independently screened titles and abstracts based on the inclusion and exclusion criteria. Finally, studies that provide reference data needed by this meta-analysis were selected through full-text reading, and discussions with a third author (BXG) were conducted when disagreements occurred between the two researchers (LLL and YSG).

### 2.2 Study selection

Original studies eligible for inclusion in this meta-analysis had to meet the following criteria:(1) being limited to English articles, (2) studies investigating the TLSs *in situ* in tumor tissue by applying immunohistochemistry and H&E staining; (3) studies focusing on evaluating the prognostic value of TLSs in cancer patients treated with ICIs, and (4) reporting clinical outcomes such as overall survival (OS), progression-free survival (PFS), objective response rate (ORR). The exclusion criteria were as follows: (1) conference abstracts, letters to the editor, reviews, comments, and animal trials; (2) studies with sample sizes < 20, since a small sample size induces publication bias; and (3) work without raw data that could be traced.

### 2.3 Data extraction

The data extracted from the included studies encompassed various variables, including the publication year, name of the first author, region, the type of ICIs, the type of tumor, TLS detection methods, number of enrolled patients, clinical outcome measures, the radio of sex, Newcastle-Ottawa scale and type of study (**Table 1**). The hazard ratios (HRs) and their associated 95% confidence intervals (95% CIs) from univariate or multivariate analysis were extracted. The assessment of each study was conducted by two authors (LLL and GYS) independently using the Newcastle-Ottawa scale (NOS), ranging from 0 to 9. Studies with an NOS score ≥ 6 were classified as high-quality studies. The two authors independently conducted the process and had discussions with a third author (BXG) when disagreements occurred.

**Table 1:** Characteristics of included studies.

Year	Author	Region	Treatment	Tumor	Cut-off criteria	Number of patients	Outcome	Gender (male/fem ale)	Newcastle-Ottawa scale	Study design
2023	Gavrielatou N	USA	Nivolumab	HNSCC	Presence	50	PFS;OS	/	9	P
2023	Hayashi Y	Japan	Nivolumab	EC	Density	34	ORR;PFS	27/7	8	R
2023	Komura K	Japan	Pembrolizumab	UC	Presence	100	PFS;OS;ORR	78/22	7	R
2022	Jieqiong Liu	China	Camrelzumab/apatinib	TNBC	Mean area	34	PFS;ORR	/	8	P
2020	Wenhao Xu	China	TKIs/ICIs	ccRCC	Degree of maturity	230	ORR	/	8	R
2020	Rita Cabrita	Sweden	anti-CTLA4/anti-PD1	Melanoma	Signature score	/	OS	/	8	R
2019	Tuba N Gide	Australia	Nivolumab/Pembrolizumab/ Ipilimumab	Melanoma	Signature score	/	OS	/	8	R
2017	Riaz N	USA	Nivolumab	Melanoma	Signature score	/	OS	/	8	P
2015	Van Allen EM	USA	Ipilimumab	Melanoma	Signature score	/	OS	/	8	R

HNSCC, head and neck squamous cell carcinoma; EC, esophageal cancer; UC, urothelial carcinoma; TNBC, triple-negative breast cancer; ccRCC, clear cell renal cell carcinoma; PFS, progression-free survival; OS, overall survival; ORR, objective response rate; P, Prospective; R, Retrospective.

### 2.4 Statistical analyses

Statistical software R software (version 4.1.0) was used to perform the analysis, while HR and 95% CI data were log transformed and pooled. Before performing a meta-analysis containing studies with different types of cancer and different types of immune checkpoint inhibitors (ICIs), heterogeneity was assessed through the implementation of a chi-square test and the I^2^ metric. The I^2^ value serves as an indicator of the proportion of variability across the pooled estimates that can be attributed to statistical heterogeneity. Studies with an I^2^ value exceeding 50% were considered to exhibit significant heterogeneity, where a random effects model was used for the analysis. Potential sources of heterogeneity were identified through the utilization of Baujat plots, and subsequent sensitivity analyses were carried out by systematically excluding individual studies. While a fixed effects model was utilized when heterogeneity was low (I^2^<50%), where sensitivity analysis is not required. Subsequently, the forest maps were subsequently created, followed by a comprehensive description of the pooled HR or OR accompanied by its corresponding 95% confidence interval (CI). Subgroup analyses of OS, PFS and ORR were performed based on patients’ characteristics. Publication bias was evaluated by an inverted funnel plot and was quantified by Egger’s and Begg’s tests. A two-sided α of less than 0.05 was considered statistically significant.

## 3 Results

### 3.1 Study selection and characteristics

**Figure 1:**
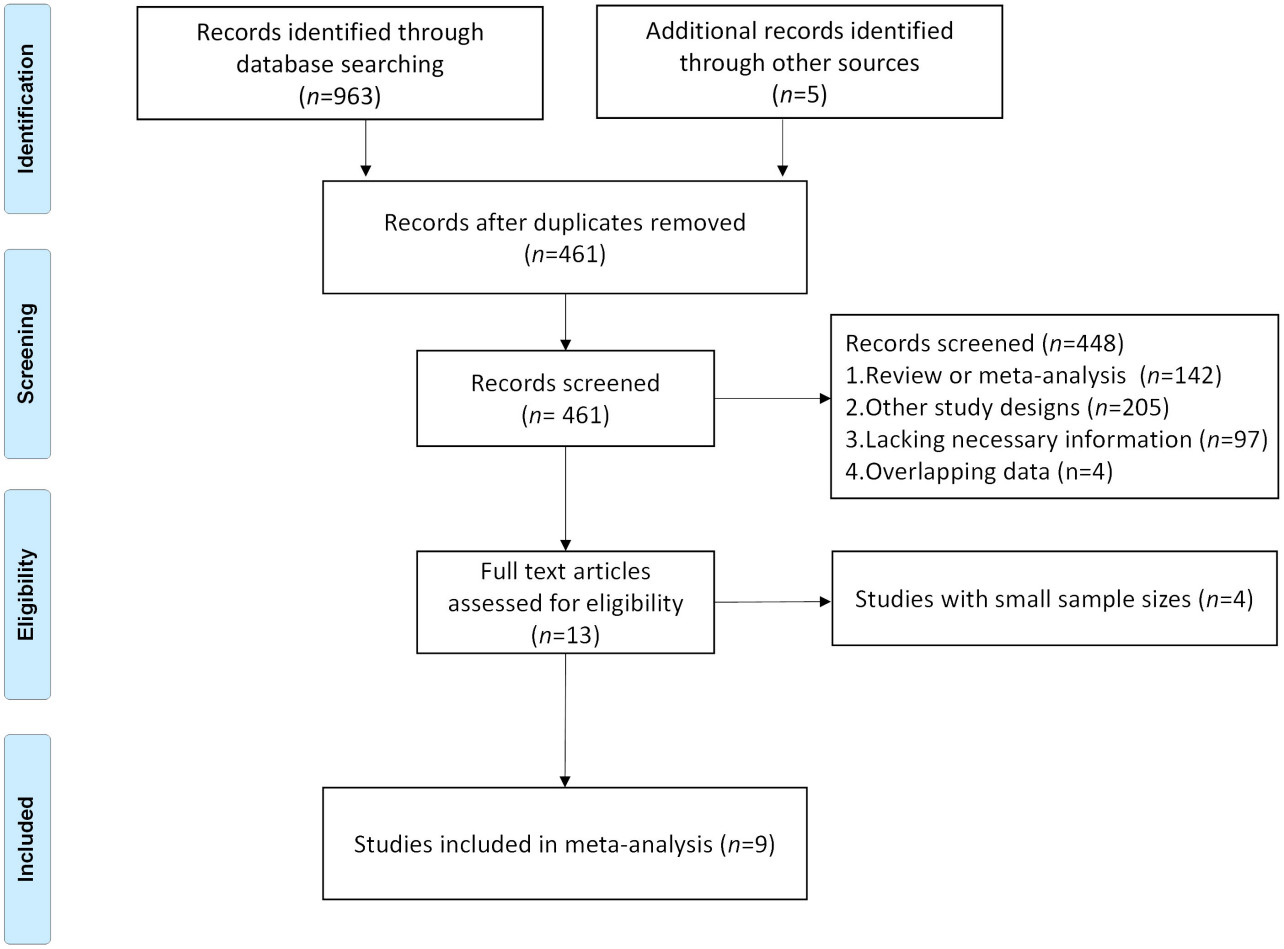
Flow diagram of study selection for inclusion in this meta-analysis.

Using the described search strategy, a total of 461 non-duplicated studies were identified. After screening based on predetermined criteria, 13 studies were selected for further evaluation through abstract review in accordance with the inclusion criteria. Due to small sample sizes, the 4 studies (14, 17–19), were only included in the systematic review. Therefore, this meta-analysis selected 9 studies (11–13, 15, 16, 21, 28–30) investigating the correlation between TLSs and clinical outcomes in cancer patients treated with immune checkpoint inhibitors. **Figure 1** shows the flow diagram for literature retrieval and selection.

The basic information and main characteristics of the 9 included studies were shown in **Table 1**. Among the 9 included studies, six cohorts had a retrospective study design, and three cohorts were prospectively designed or from prospective trials (12, 21, 29). Among the 9 included studies evaluating the prognostic value of TLSs in cancer patients treated with ICIs: 6 studies evaluated overall survival (OS) (11, 12, 21, 28–30), 3 studies evaluated progression‐free survival (PFS) (13, 16, 21), and 4 studies evaluated objective response rate (ORR) (12, 13, 15, 16). All the studies included in the analysis obtained moderately high scores on quality assessments conducted using the Newcastle-Ottawa Scale. Out of the 9 studies that were included, 4 studies focused on specific types of cancer, with melanoma being the most commonly reported tumor (11, 28–30). The ICIs used in the aforementioned studies included PD-1 antibody (pembrolizumab and nivolumab were commonly used), PD-L1 antibody, CTLA-4 antibody, and combination therapies including immunotherapy.

TLSs were divided into high levels and low levels based on different cut-off criteria. Among the 9 studies included in this analysis, different cut-off criteria corresponded to different HR. In the subsequent investigation of the relationship between TLSs and OS, PFS, and ORR, we established inclusion criteria. If a study employs two distinct TLS cut-off criteria, our preference was given to the hazard ratio (HR) associated with Density, Degree of maturity, or Maximal diameter. In cases where a study applies both Density and Degree of maturity, or Density and Maximal diameter for TLS grading, the HR linked to Density was the one we chose.

### 3.2 Prognostic value of TLSs in cancer patients treated with ICIs

**Figure 2:**
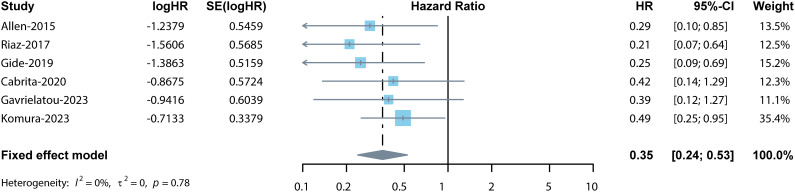
Forest plot for the association between TLSs and OS. The result showed the high levels of TLSs predicted a significantly prolonged OS (pooled HR = 0.35, 95% CI: 0.24–0.53, p < 0.001) in cancer patients treated with ICIs.

Among the 9 studies selected for this meta-analysis, 6 studies reported the association between OS and TLSs (11, 13, 21, 28–30). Considering the low heterogeneity (I^2^ = 0%), a fixed effects model was used for analysis. Results indicated the pooled HR was 0.35 (95% CI: 0.24–0.53, p < 0.001, **Figure 2**), suggesting the prognostic role of TLSs in cancer patients treated with ICIs.

**Figure 3:**
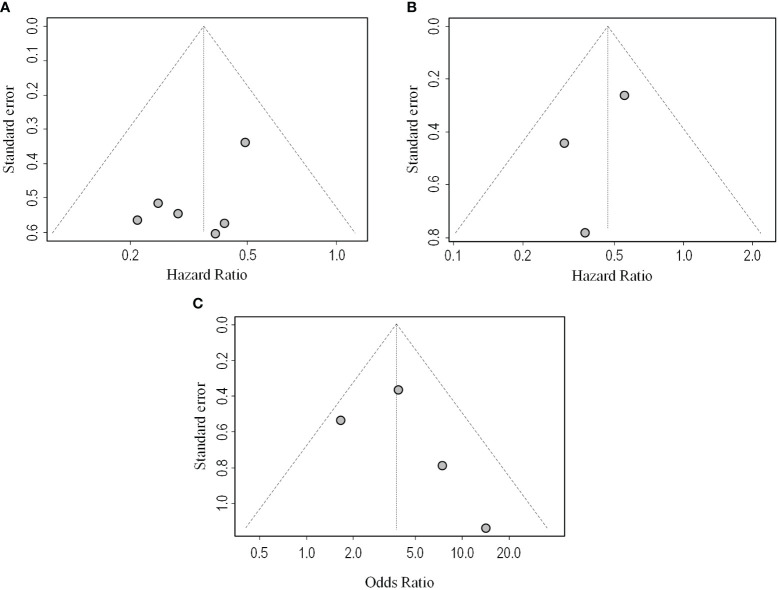
Funnel plots of OS **(A)**, PFS **(B)**, ORR **(C)**. Funnel plots for checking potential publication bias. The funnel plots exhibited approximate symmetry **(A–C)**, indicates that no publication bias occurs in the meta-analysis results.

The sensitivity analysis confirmed the pooled results’ credibility and stability. The funnel plots exhibited approximate symmetry (**Figure 3A**). Egger’s and Begg’s tests showed no significant publication bias (p = 0.154, p = 0.851), indicating that high levels of TLSs significantly predicted prolonged OS in cancer patients treated with ICIs.

**Figure 4:**
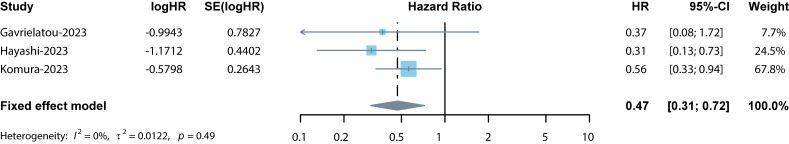
Forest plot for the association between TLSs and PFS. The result showed the high levels of TLSs predicted a significantly prolonged PFS (pooled HR = 0.47, 95% CI: 0.31–0.72, p < 0.001) in cancer patients treated with ICIs.

Among the 9 studies selected for the meta-analysis, 3 studies reported the association between PFS and TLSs (13, 16, 21). Considering the low heterogeneity (I^2^ = 0%), a fixed effects model was used for analysis. Results indicated the pooled HR was 0.47 (95% CI: 0.31–0.72, p < 0.001, **Figure 4**). The sensitivity analysis confirmed the pooled results’ credibility and stability. The funnel plots exhibited approximate symmetry (**Figure 3B**). Egger’s and Begg’s tests showed no significant publication bias (p = 0.489, p = 0.602), indicating that high levels of TLSs significantly predicted better PFS in cancer patients treated with ICIs.

**Figure 5:**
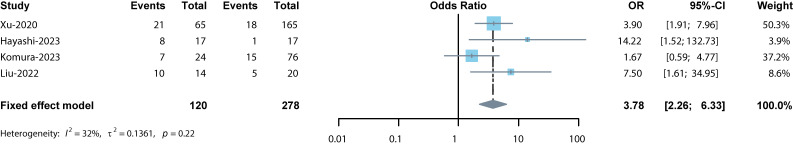
Forest plot for the association between TLSs and ORR. The result showed the high levels of TLSs predicted a significantly lower ORR (pooled OR = 3.78, 95% CI: 2.26–6.33, p < 0.001) in cancer patients treated with ICIs.

The ORR was reported in 4 studies which included 398 cancer patients treated with ICIs (12, 13, 15, 16). The pooled OR was 3.78 (95% CI: 2.26–6.33, p < 0.001, I^2^ = 32%, **Figure 5**) suggesting that TLSs were associated with lower tumor response. Similar results were obtained after conducting a sensitivity analysis. The funnel plots exhibited approximate symmetry (**Figure 3C**). Egger’s and Begg’s tests showed no significant publication bias (p = 0.478, p = 0.174).

## 4 Discussion

In recent years, the role of TLSs in immunotherapy response to solid tumors has received extensive attention (11–19, 21, 28–31), with melanoma being the most commonly reported tumor (11, 17, 28–30). In general, the presence or a higher density of TLSs were predicting a significantly prolonged OS and PFS in cancer patients treated with ICIs. Notably, a few studies also reported TLS presence was not significantly associated with either PFS or OS in cancer patients treated with ICIs, such as head and neck squamous cell carcinoma and colorectal cancer (20, 21). Hence, we conducted this meta-analysis in an effort to comprehensively summarize the prognostic significance of TLSs in cancer patients treated with ICIs.

Thus far, this study included 9 studies and represented the largest meta-analysis comprehensively summarizing the prognostic value of TLSs in cancer patients treated with ICIs. Although two previous meta-analysis reported the prognostic value of TLSs in cancer patients treated with ICIs (20, 32), they only included gastrointestinal cancers or breast cancer. A more extensive literature search was done in this study and we included more data for various tumors and built a good basis for evaluating the prognostic value of TLSs in cancer patients treated with ICIs. Moreover, sensitivity analyses and Baujat plots were conducted to validate the stability of the obtained results. The clinical outcomes of our results included OS, PFS and ORR. A present or higher density of TLSs significantly predicted prolonged OS and PFS, and better tumor response in cancer patients treated with ICIs, which suggested that high levels of TLSs could predict a favorable prognosis for cancer patients treated with ICIs.

Our results indicated that high levels of TLSs could predict a favorable prognosis for cancer patients treated with ICIs and have the potential to become a prognostic biomarker of immunotherapy-related patients. Our study represents a significant contribution to the field, offering valuable implications for future clinical practice. Given the cost of immunotherapy and the potential for drug toxicity, clinician needed to screen which cancer patients are suitable for immunotherapy. Tertiary lymphoid structure is a readily available biomarker that can be obtained directly by HE staining of biopsy or excision samples, and its lower cost makes it more easily applicable in different clinical scenes and countries with different incomes.

However, this meta-analysis had several limitations. Firstly, there is a lack of uniform standards for TLSs evaluation. The diversity in these classification methods may influence the prognostic predictive power associated with TLSs. Secondly, part of the included studies are retrospective studies, which may lead to inevitable selection bias and confounding bias, but Baujat plots and sensitivity analyses were subsequently performed to validate the stability of results. Thirdly, there were fewer studies included in certain analyses, especially in the association between PFS and TLSs, with only 3 immunotherapy studies included, which may affect the evaluation of the role of TLSs in prognosis. Fourthly, this study only included data related to intratumoral TLSs, which may not fully reflect its predictive role in prognosis. These limitations can affect the interpretation of the identified significant associations between high levels of TLSs and clinical outcomes in cancer patients treated with ICIs. However, the results of this study were reliable because low heterogeneity was detected and publication bias was not observed among most of the results.

## 5 Conclusions

Our results indicated that high levels of TLSs could predict a favorable prognosis for cancer patients treated with ICIs and have the potential to become a prognostic biomarker of immunotherapy-related patients.

## Data availability statement

The original contributions presented in the study are included in the article/supplementary material. Further inquiries can be directed to the corresponding author.

## Author contributions

LL: Writing – review & editing. YG: Writing – original draft. BG: Writing – original draft. SW: Writing – original draft. MW: Writing – original draft. PS: Writing – original draft. SJ: Writing – original draft. LY: Writing – original draft, Writing – review & editing.
